# Why verifying diagnostic decisions with a checklist can help: insights from eye tracking

**DOI:** 10.1007/s10459-015-9585-1

**Published:** 2015-02-12

**Authors:** Matthew Sibbald, Anique B. H. de Bruin, Eric Yu, Jeroen J. G. van Merrienboer

**Affiliations:** 1 Department of Medicine, McMaster University Hamilton General Hospital, McMaster Wing Room 600, 237 Barton St, Hamilton, ON L8L2X2 Canada; 2 Department of Medicine, Faculty of Medicine, University of Toronto, Toronto, Canada; 3 Department of Educational Development and Research, Faculty of Health, Medicine and Life Sciences, School of Health Professions Education, Maastricht University, Maastricht, The Netherlands

**Keywords:** Checklists, ECG interpretation, Clinical reasoning, Cognitive load

## Abstract

Making a diagnosis involves ratifying or verifying a proposed answer. Formalizing this verification process with checklists, which highlight key variables involved in the diagnostic decision, is often advocated. However, the mechanisms by which a checklist might allow clinicians to improve their verification process have not been well studied. We hypothesize that using a checklist to verify diagnostic decisions enhances analytic scrutiny of key variables, thereby improving clinicians’ ability to find and fix mistakes. We asked 16 participants to verify their interpretation of 12 electrocardiograms, randomly assigning half to be verified with a checklist and half with an analytic prompt. While participants were verifying their interpretation, we tracked their eye movements. We analyzed these eye movements using a series of eye tracking variables theoretically linked to analytic scrutiny of key variables. We found that more errors were corrected using a checklist compared to an analytic prompt (.27 ± .53 errors per ECG vs. .04 ± .43, *F*_1,15_ = 8.1, *p* = .01, *η* ^2^ = .20). Checklist use was associated with enhanced analytic scrutiny in all eye tracking measures assessed (*F*_6,10_ = 6.0, *p* = .02). In this experiment, using a key variable checklist to verify diagnostic decisions improved error detection. This benefit was associated with enhanced analytic scrutiny of those key variables as measured by eye tracking.

## Introduction

Making a diagnosis involves ratifying or verifying a proposed answer. This verification process, where errors are sought out and corrected, is common to all professionals. It is an important part of what Donald Schön describes as reflection ‘in practice’ where professionals dynamically make adjustments to their decision making in real time (Schön 1983). In the hypothetic deductive model, clinicians test their diagnostic hypothesis before adopting it (Elstein et al. 1978). In a system-processing model, clinicians must weigh diagnostic suggestions from analytic and non-analytic processing, in what some have hypothesized is a ‘calibrator’ (Croskerry 2009). In the illness script model, clinicians assess the fit of their diagnosis to the clinical situation (Charlin et al. 2000). Recent interest has focused on how this verification process can be improved, to increase the chance that clinicians can spot and correct errors when verifying diagnostic decisions. One such approach is the use of checklists as popularized by Gawade in his book “Checklist manifesto” (2009) and reviewed by Ely et al. (2011).

Prior study suggests that using checklists to review diagnostic decisions reduces clinicians’ errors, both in visual test interpretation such as electrocardiograms (ECGs) and in physical exam diagnosis of common valvular diseases (Sibbald et al. 2013a, b, 2014). The relative benefit of this approach persists regardless of level of expertise, though its absolute magnitude is attenuated among experts who have fewer errors to correct (Sibbald et al. 2014). Why might there be benefit? Gawade (2009) suggests that when clinicians diagnose, they weigh different variables: symptoms, signs or investigations. The diagnosis is a summative decision, developed from key information. When clinicians verify these diagnostic decisions, using a list of relevant variables can help them focus on relevant rather than distracting details potentially improving error detection. In one study, clinicians reviewing their ECG interpretations were better able to spot errors using a checklist comprised of key variables (i.e., ECG rate, rhythm, axis, evidence of hypertrophy, ischemia and abnormal intervals) than when receiving an analytic prompt asking them to simply “reinterpret this ECG trying your best to correct your own mistakes” (Sibbald et al. 2013).

We hypothesize that checklists can direct clinicians to scrutinize more carefully those details which are known to be most relevant. In a study of cardiac physical exam using a high fidelity simulator, clinicians could find and fix up to 10 % of their diagnostic errors by reviewing their diagnoses with a key variable checklist (Sibbald et al. 2013). However, verifying the decision with a checklist only helped when physicians could re-examine the simulator. Simply verifying key variables from memory using a checklist was insufficient. Clinicians had to re-collect or re-perceive these key variables to spot errors.

The purpose of this study was to explore the hypothesis that key-variable checklists aided clinicians in verifying diagnostic decisions by increasing analytic re-examination of key variables. We chose to study this using eye tracking to compare re-examination of ECG diagnostic decisions with and without the use a key variable checklist (i.e., rate, rhythm, axis, evidence of hypertrophy, ischemia and abnormal intervals).

## Eye movements and ECG interpretation

To better understand why eye movements might give insight into what clinicians do when they verify a diagnosis, a brief description of eye movements and ECG composition is necessary. Eye movements are frequently divided into periods of time where the eye rests in a fixed place, termed fixations, and periods of time where the eye rapidly moves from one place to another, termed saccades (Holmqvist et al. 2011). During saccades, no information is registered or interpreted.

ECGs are pictographic representations of the electrical activation of the heart. Electrical activation spreads through different parts of the heart in different stages resulting in a series of waves. These waves are captured from 12 different locations or leads simultaneously. The recording of the ECG occurs over 10 s capturing an average of 6–17 activations of the heart when the heart rate is normal. Usually, the ECG is displayed as a twelve box grid containing 2.5 s of each lead, with a rhythm strip which displays the full 10 s of one of these twelve leads.

When verifying an ECG interpretation, careful scrutiny of key variables is likely to increase verification time and verification eye movement patterns. An increase in verification time is likely to result in more fixations, more saccades and a greater sum of all these saccades (i.e., scan path). Scrutiny is also likely to change the pattern of eye movements. Enhanced scrutiny should increase the proportion of saccades within a lead compared to between leads, as clinicians re-collect or re-perceive key variables related to the relationship between waves (for instance, understanding the rhythm requires scrutiny of the initiating “P” wave morphology and its subsequent relationship to the “QRS” complexes). Similarly, more scrutiny is likely to increase the ratio of short “local” to long “global” saccades, in contrast to the predominant “global” saccades reported when experts make gestalt judgments (Zangemeister et al. 1995). Finally, more scrutiny is likely to increase the percentage of the 12 leads checked.

Therefore, if verifying ECGs with a checklist compared to analytic prompt increases the analytic re-examination of key variables we hypothesize that eye-tracking will show increased checking time, number of fixations, scan path length, ratio of intra- to extra-lead saccades, ratio of local to global saccades, and proportion of the 12 leads with at least one fixation.

## Method

### Participants

16 cardiology residents with 4–6 years of post-graduate experience interpreting ECGs were recruited by email. This was a convenience sample derived from 31 residents in the post-graduate cardiology program.

## Material

2 ECGs were selected from the difficult section of an online repository of ECGs (www.ECGmadesimple.com). ECGs were analyzed by two experts, and selected for having an unambiguous and agreed upon interpretation.

### Protocol

For each ECG, participants were asked to sequentially: (1) interpret the ECG, (2) verify their interpretation with either checklist or analytic prompt determined at random using a within-subject design, and (3) rate the cognitive load required to verify their interpretation. Each step is explained in detail below.

1. ECGs were presented using an electronic experiment suite (SMI Experiment Centre, SensoMotoric Instruments GmbH, Teltow). Residents were asked to “Interpret each ECG as you ordinarily would. Do not share your thoughts as you interpret. Verbally report only your final interpretation”. Residents were discouraged from thinking aloud. If residents started to provide verbal explanations, they were interrupted and the task re-iterated.
2. Residents were asked to verify their interpretation in one of two conditions: checklist (CH) and analytic prompt (AP). The analytic prompt was “Silently check your interpretation. When you are ready, verbally report ‘no change’ or ‘change my interpretation to’”. The instruction for the checklist was: “Silently check your interpretation by systematically identifying each of the following: rate, rhythm, axis, hypertrophy, ischemia, intervals. When you are ready, verbally report ‘no change’ or ‘change my interpretation to:’”. Residents were asked to check half of the ECGs (i.e., 6) in each condition. Instructions for each condition were given on a separate screen before each ECG interpretation could be verified. The order of the ECGs was kept constant, while the order of the conditions was randomized for each participant. Eye movements were tracked while residents verified their interpretation.

### Analysis

The number of errors was counted in each interpretation and subsequent verification with either the checklist or analytic prompt. Each ECG contained between 1 and 5 diagnoses. Each missing diagnosis was counted as an error as well as any additional incorrect diagnoses given. Reduction in errors was calculated as the difference between the numbers of errors in the reinterpretation compared to the initial interpretation. For each subject, errors were averaged for all ECGs in each of the two verification conditions.

Eye movements were analyzed using BeGaze 2.4 (SensoMotoric Instruments GmbH, Teltow). All analyses were preplanned, including the selection of eye tracking variables to avoid post hoc biases. To identify intra versus extra-lead saccades, areas of interest (AOIs) were constructed for each ECG lead and four equal areas of the rhythm strip that runs along the bottom of the ECG in BeGaze. Each AOI was exactly the same size, and in the same location for all 12 ECGs. AOIs were constructed before data collection to avoid post hoc bias. The local to global saccade ratio was defined as the number of saccades <1.1° divided by the number of saccades >1.1° (Zangemeister et al. 1995). Pupil diameter was calculated by a proprietary algorithm in BeGaze. Saccadic rate was calculated as the number of saccades per second. The summed fixation time within each AOI was averaged to calculate the average dwell time.

Four repeated ANOVAs were conducted. Checking condition was used as within subject variable. Measures included reduction in errors (analysis 1), and the six eye-tracking variables hypothetically linked to analytic behaviors (analysis 2, see Table 1 for the eye-tracking variables).

**Table 1:** Influence of checklists on eye tracking variables related to enhanced analytic scrutiny during diagnostic verification

Measure	Hypothesis comparing checklists (CH) versus analytic prompts (AP)	Data				
		Verification with checklist *µ* ± *SD*	Verification with analytic prompt *µ* ± *SD*	*F*_1,15_	*p*	*η* ^2^
Task time (seconds)	CH > AP	32 ± 21	21 ± 15	11.8	.004	.24
Number of fixations	CH > AP	88 ± 71	52 ± 37	16.8	.001	.23
Scan path length (megapixels)	CH > AP	13.1 ± 8.8	7.9 ± 4.8	12.4	.003	.23
Ratio of intra to extra lead saccades	CH > AP	8.1 ± 5.4	6.1 ± 4.5	10.0	.006	.07
Ratio of local to global saccades (i.e. saccades <1.1° vs. >1.1°)	CH > AP	.37 ± .12	.33 ± .14	6.5	.02	.04
Proportion of the 12 leads with at least one fixation	CH > AP	.65 ± .20	.55 ± .23	21.3	.001	.08

All statistics were done in SPSS version 20 (IBM, Redmond). All variables are reported as means (*µ*) ± standard deviation (*SD*). A *p* value of <0.05 was treated as significant. Eta squared (*η* ^2^) values were calculated to estimate effect sizes. Effects were considered small, medium and large based on cutoff values of .01, .059 and .138 respectively.

Ethical approval was obtained from the University of Toronto ethics review board.

## Results

Participants made an average of 1.5 errors per ECG (*SD* = 1.4). When verifying their decision, participants corrected an average of .16 mistakes per ECG (*SD* = .50). The number of errors identified differed by checking condition (within-subject main effect *F*_1,15_ = 8.1, *p* = .01, *η* ^2^ = .20). Only checklist use was associated with a significant number of errors being corrected (.27 ± .26 errors per ECG with checklist use versus .04 ± .21 with the analytic prompt).

**Fig. 1:**
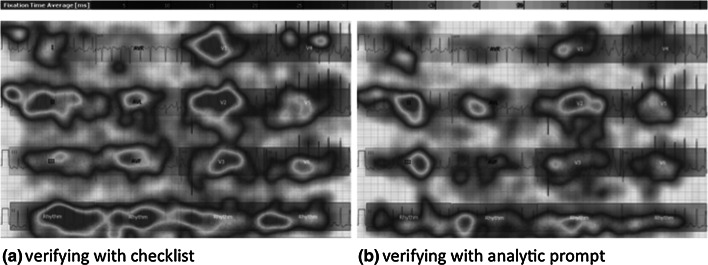
Average fixation time when verifying ECG interpretations. Heat map of the average fixation time (in milliseconds) for all participants and ECGs. *Blue hues* represent areas which were attended to sparingly (<15 ms), whereas *red hues* represent areas with longer fixation times (>60 ms). Areas of interest are labeled (e.g., I, II, III etc.). For each area of interest, longer fixation times (*more red hues*) are seen when verifying with a checklist (**a**) compared to verifying with an analytic prompt (**b**). (Color figure online)

Verifying ECG interpretations with a checklist was associated with analytic re-examination of key variables as assessed by eye-tracking (within-subject main effect *F*_6,10_ = 6.0, *p* = .02). As hypothesized, checklist use was associated with longer verification times, greater number of fixations, longer scan paths, greater ratio of intra to extra-lead saccades, greater ratio of local to global saccades and greater proportion of 12 leads with at least one fixation (all *p*’s < 0.02, Table 1). Effect sizes varied, with the checklist condition having large effects in prolonging checking time, increasing the number of fixations and lengthening the scan path (upper part of Table 1). To graphically display the differences in eye movements between conditions, heat maps were constructed by averaging the fixation time of all participants for all ECGs. Qualitatively, use of checklists was associated with greater fixation times in all leads, represented as red hues in Fig. 1.

## Discussion

This study provides evidence that eye-tracking technology can be used to gain insight into how clinicians verify their diagnostic decisions. While eye tracking has been used to assess ECG interpretation skills, our adaptation of the technology to study decision-making is novel. Previous studies have focused exclusively on the differences between novices and experts (Augustyniak 2003; Augustyniak and Tadeusiewicz 2006). Application of eye tracking to study decision making may be helpful in many other domains of medicine, particularly in visual specialties like radiology and pathology.

The salient findings were:

1. Verifying diagnostic decisions with a checklist was helpful. Participants were more likely to find and fix errors when verifying with a checklist compared with an analytic prompt. While the benefit was small, its magnitude is similar to prior reports (Sibbald et al. 2013a, b, 2014). We argue that even a small effect is likely to be clinically meaningful with little downside. Participants corrected 1 error out of every 6 ECGs they checked, by spending an additional 30 s. While the clinical relevance of errors was not directly assessed, some involved life-threatening diagnoses including acute infarction and ventricular tachycardia.
2. Verifying diagnoses with a checklist resulted in more analytic scrutiny of key variables, as assessed by eye tracking measures. Verification was more thorough—participants spent more time and checked more places (i.e., greater number of fixations, scan path lengths and percentage of leads re-examined). Checklist use was also associated with more detailed scrutiny of the ECG (i.e., greater proportion of local to global and intra lead to extra lead saccades). This suggests that participants were re-examining individual components of the ECG, the primary data involved in interpretation decisions, potentially addressing errors related to premature diagnostic closure.

These findings have significant implications for clinicians and teachers. Analytic scrutiny is likely important when diagnostic decisions are verified. Clinicians should focus on scrutinizing key variables, as promoted by the checklist used in this study. When designing checking tools, efforts should be focused on promoting analytic scrutiny, either through increasing time or thoroughness of the verification process. These findings also have important implications for teaching. While emphasizing intuition may be helpful in learning or deciding (Ark et al. 2006; Eva et al. 2007; Kulatunga-Moruzi et al. 2001), verifying those decisions is a different skillset. Teach that verification benefits from being structured and analytic.

We recognize that using a key variable checklist to improve decision verification requires identification of diagnostic task where the problem space is well defined and key variables can be identified (e.g., ECG interpretation, chest X-ray interpretation, or cardiac physical exam). However, diagnostic tasks where the key variables cannot be easily predicted (e.g., an office visit for general fatigue) are ill suited to this approach. In these situations, methods to improve reflective practice may prompt the same type of cognitive processes via a different approach. For instance, previous studies have asked clinicians to reflect on their proposed diagnosis using backwards reasoning to re-examine it (e.g., If your diagnosis is correct, what key features should be present but are missing? What key features suggest an alternate diagnosis?) (Mamede and Schmidt 2004; Mamede et al. 2008; Mamede et al. 2007). Clinicians are still asked to reconsider key variables. However, they must decide for themselves what key variables to consider. Further study exploring the selective advantages to using key variable checklists or promoting reflective practice is warranted to help clinicians identify situations that are better suited to each approach.

Several limitations are worth highlighting. First, our study participants all had an intermediate level of expertise at ECG interpretation. Experts and novices might use checklists differently to verify their interpretations. This is a worthy area for future study. Second, we did not standardize time spent on verifying the decision. The benefit seen with checklists could be entirely related to the increased time spent. However, differences in eye tracking variables, such as the ratio of intra to extra lead saccades, suggest that checklist use modified what participants were doing, as well as prolonging how long they took to check. Further study could limit time allotted for verification with and without checklists to determine if prolonging verification time is critical to checklist benefit. Last, we had no control over what participants actually did. Those given an analytic prompt could have used a formal approach resembling a checklist, which would have moderated differences between the two strategies.

In conclusion, we note that eye tracking is a promising research tool for furthering our understanding of diagnostic decision making. Use of eye tracking supports the concept that verifying diagnostic decisions with a checklist is helpful because it enhances analytic scrutiny of key variables.
